# Randomised controlled feasibility trial of retroperitoneal vs transperitoneal robot‐assisted partial nephrectomy: the ROPARN study

**DOI:** 10.1111/bju.16653

**Published:** 2025-01-22

**Authors:** Sebastian Kälble, Simon U. Engelmann, Hannah Schrutz, Florian Zeman, Emily Rinderknecht, Maximilian Haas, Christoph Pickl, Christopher Goßler, Yushan Yang, Stefan Denzinger, Maximilian Burger, Johannes Bründl, Roman Mayr

**Affiliations:** ^1^ Department of Urology, St. Josef Medical Center University of Regensburg Regensburg Germany; ^2^ Center for Clinical Studies University Hospital Regensburg Regensburg Germany

**Keywords:** nephron‐sparing surgery, randomised controlled study, retroperitoneoscopic surgery, retroperitoneal, renal neoplasm, transperitoneal, transperitoneoscopic surgery, kidney surgery

## Abstract

**Objectives:**

To assess the feasibility of trial recruitment and confirm that retroperitoneal robotic partial nephrectomy (RRPN) has the same oncological efficacy as transperitoneal robotic partial nephrectomy (TRPN), with time advantages and less peri‐operative morbidity, in a randomised controlled trial (RCT).

**Patients and Methods:**

The study was designed as a single‐centre, open‐label, feasibility RCT. Patients with suspected localised renal cell carcinoma referred for robotic partial nephrectomy were randomised in a 1:1 ratio to receive either TRPN or RRPN. The primary outcomes were trial feasibility, postoperative mobility and pain perception. Secondary outcomes were intra‐operative times, assessment of complications, and comparison of positive surgical margin (PSM) rates. The data on all randomised patients who underwent surgery were analysed descriptively.

**Results:**

Sixty‐one patients underwent TRPN or RRPN (31 vs 30). Postoperative mobility within 24 h after surgery (RRPN: 77 vs TRPN: 71%; *P* = 0.613), median postoperative pain, assessed using a numeric rating scale (RRPN: 1.5 vs TRPN: 1.8; *P* = 0.509), and full bowel canalisation within 3 days (RRPN: 100% vs TRPN: 90%; *P* = 0.315) were more favourable in the RRPN group, but the difference was not statistically significant. In comparison to TRPN, RRPN was associated with shorter operating time (81 vs 105 min; *P* = 0.005), shorter time on the console (49 vs 73 min; *P* < 0.001) and shorter time from console to renal artery preparation (7.5 vs 18 min; *P* < 0.001). TRPN required a shorter time from skin incision to console (16 vs 12 min; *P* = 0.001). There was no statistically significant difference in tumour complexity, ischaemia time, PSM rate, blood loss or complications between the two groups.

**Conclusion:**

We present the first data from an RCT comparing RRPN with TRPN. RRPN showed significant time benefits while being a safe alternative to TRPN, with a similar PSM rate. There was less postoperative morbidity in the RRPN group, although this was not statistically significant. These results underline that dorsolateral renal tumours should be preferably resected by RRPN. Further multicentre RCTs are necessary to confirm these results.

**Abbreviations**
IQR: interquartile range
JH‐HLM: John Hopkins Highest Level of Mobility
LOS: length of inpatient stay
PSM: positive surgical margin
RCT: randomised controlled trial
RPN: robotic partial nephrectomy
RRPN: retroperitoneal robotic partial nephrectomy
TRPN: transperitoneal robotic partial nephrectomy

## Introduction

Robotic partial nephrectomy (RPN) has become the standard treatment for patients with localised RCC [1, 2]. Since its first description in 2004, RPN has replaced conventional laparoscopic partial nephrectomy as the predominant minimally invasive approach [3, 4, 5]. RPN can be performed via transperitoneal or retroperitoneal access. While the transperitoneal cavity is a predefined anatomical space, the retroperitoneal space needs to be established with balloon dilatation and peritoneum mobilisation [6]. As a result, the working space in the retroperitoneum is small and narrow, which presents greater difficulty for surgeons and is reflected in a longer learning curve [7, 8]. By contrast, the retroperitoneal approach provides quick access to the dorsolateral face of the kidney, including the renal artery, and no abdominal organs need to be mobilised to access the kidney [9]. In addition, in patients who have previously undergone abdominal surgery, retroperitoneal access can avoid intra‐abdominal adhesions and potential bowel complications [10, 11]. Advantages of the transperitoneal approach are easy access, the rigorous working space, uncomplicated port placement and the possibility to perform any kind of kidney surgery. Disadvantages are organ mobilisation, the dorsal location of the renal artery, and kidney mobilisation for RPN [9]. However, only a small proportion of robotic kidney surgeons have retroperitoneal skills and therefore most RPNs are performed using a transperitoneal approach. The choice between the two approaches is usually based on the surgeon's skills, preference, and tumour localisation. For surgeons skilled in the retroperitoneal approach, tumours with dorsolateral location are preferably operated on using retroperitoneal RPN (RRPN). Upper pole tumours can be challenging if they are complex and have an anterior location [12]. RRPN is feasible for lower pole tumours even  when the renal mass involves the ventral part of the kidney [13]. In addition, patients with prior abdominal surgery or with a hostile abdomen are good candidates for retroperitoneal access. Although there are several interesting aspects of RRPN, the procedure is underrepresented in the field [8].

Retrospective studies have shown that RRPN may provide advantages in terms of shorter operating and warm ischaemia times, less estimated blood loss, and less requirement for postoperative analgesics [10, 14, 15, 16]. In tumours with a posterior location these benefits are increased [15]. Unfortunately, the available studies comparing the retroperitoneal and transperitoneal approaches for RPN are retrospective and are therefore at risk of selection, information and recall bias. To date, there is no available evidence from randomised controlled trials (RCTs) comparing the two surgical approaches.

To overcome the limitations of retrospective studies comparing RRPN with transperitoneal RPN (TRPN), we conducted a prospective single‐centre RCT. A further important rationale for conducting an RCT comparing RRPN with TRPN is to generate robust data to plan an adequately powered multicentre RCT in the future. This trial was conducted in accordance with the CONSORT (CONsolidated Standards Of Reporting Trials) criteria checklist (Fig. S1).

## Patients and Methods

Following ethics approval from the Ethics Committee in Regensburg (22‐2918‐101) and registration with the German Clinical Trials Register (DRKS‐ID: DRKS00029650), recruitment began in September 2022 and continued up to November 2023. The study was designed as a single‐centre, open‐label, investigator‐initiated, interventional, feasibility RCT.

Patients with suspected RCC referred for nephron‐sparing surgery were screened and assessed for eligibility according to the criteria listed in our protocol. No biopsies were performed prior to surgery.

After meeting the inclusion criteria, each eligible patient was informed about the ongoing study and operating procedures during consultation.

After providing signed informed consent, participants were enrolled in the study by informing the study office where the randomisation process was undertaken. Randomisation concealment was ensured using a random allocation sequence, generated by the Centre for Clinical Trials, which was stored in the study office and inaccessible to the ROPARN study team. The eligibility of patients for both surgical approaches was determined by the performing surgeons. Patients with totally endophytic central tumours, extremely anterior tumours or complex anterior upper pole tumours were not eligible for both approaches. Three experienced robotic surgeons (R.M., S.D. and J.B.) performed the TRPNs (caseloads of 150, 90 and 150 TRPNs, respectively, before study commencement), whereas only one surgeon (R.M.) performed the RRPNs (30 RRPNs performed before study commencement). Each case was discussed by at least two of the three surgeons (one RRPN and one TRPN surgeon) and an experienced uroradiologist.

### Eligibility Criteria

#### Inclusion Criteria

The inclusion criteria were as follows: age ≥18 years with capacity to give consent; being scheduled for elective RPN for localised renal neoplasms; eligibility for both RRPN and TRPN, as determined using preoperative imaging; ability to understand the goal and consequences of the RCT; provision of written informed consent; and availability of recent (<90 days) CT or abdominal MRI.

#### Exclusion Criteria

The following patients were excluded: those with a history of extensive transabdominal surgery, who were therefore ineligible for TRPN; vulnerable patients (with cognitive impairments [e.g., dementia] or otherwise not legally competent); and those with ipsilateral recurrence after partial nephrectomy.

### Outcomes

The primary outcomes included feasibility of trial recruitment, pain intensity, analgesia requirement, bowel function, and postoperative mobility of the patients. Secondary outcomes were intra‐operative times, intra‐ and postoperative complications, positive surgical margin (PSM) rate and length of inpatient stay (LOS). Complications were classified using the Clavien–Dindo system [17, 18]. The intra‐operative parameters were recorded using a standardised surgical protocol, which included operating time, estimated blood loss, warm ischaemia time, time from skin incision to console, time to identification and preparation of the renal artery until clamping was possible, time on the console, and use of haemostatics. Mobilisation was quantified using the John Hopkins Highest Level of Mobility (JH‐HLM) score. Full postoperative mobilisation was defined as a JH‐HLM score of 8 (ability to walk > 75 m) [19]. Mobilisation and pain perception were recorded during ward rounds and documented prospectively in the electronic patient file. Individual mobilisation outside the patient's room was encouraged on Day 1 after surgery and was possible after surgery if patients felt confident. Pain perception was assessed using a numeric rating scale (NRS). Patients were asked about their pain three times a day (6:00 h, 14:00 h and 22:00 h). All patients received 500 mg metamizole orally four times a day on the day of surgery and on the first day postoperatively. For pain peaks patients could ask for 1 g i.v. metamizole or 5 mg oral oxycodone on demand, in accordance with the in‐house standard. If medication on demand was not sufficient, 500 mg metamizole orally four times a day was reapplied. Follow‐up was assessed 90 days postoperatively. Postoperative bowel function was assessed during ward rounds. If patients reported problems with bowel movements, laxatives could be prescribed.

### Statistical Analysis

Statistical analysis was performed using IBM SPSS Statistics Version 29.0 (IBM Corp. Armonk, NY, USA). Figures were generated in GraphPad Prism Version 10. The random allocation sequence was generated using SAS 9.4 using ‘proc plan’ by the Centre for Clinical Trials of the University Hospital Regensburg. Block‐wise randomisation using a block size of 8 was used to ensure an equal distribution of both treatments. As this was a feasibility study, no formal a priori sample size calculation was performed. Nevertheless, it should be noted that Hertzog et al. [20] recommend at least 30 participants per intervention or control group be enrolled to obtain valid direct estimates of between‐group effect sizes for subsequent power analysis in further confirmatory trials [20].

Intention‐to‐treat analysis could not be performed because, of the 64 randomised patients, only 61 underwent robotic surgery. Two randomised patients cancelled the surgery at short notice, while in one patient open partial nephrectomy was performed because of a lack of robotic surgery capacity after postponed surgery (Table S1). No interim analysis was performed. Categorial variables were reported using frequencies and proportions. Continuous variables were reported using median with interquartile range (IQR). Data were analysed using the Mann–Whitney *U*‐test for continuous variables and the chi‐squared test for categorical variables. All mentioned *P* values are two‐tailed. A *P* value less than 0.05 was taken to indicate statistical significance.

## Results

### Allocation, Demographics and Tumour Characteristics

As the ROPARN study was designed as a pilot study, one of the primary outcomes was the feasibility of trial recruitment, measured as the accrual rate. The accrual rate is the number of patients fulfilling the eligibility criteria and agreeing to participate in the RCT, divided by the number of all eligible patients within the recruitment time.

**Fig. 1:**
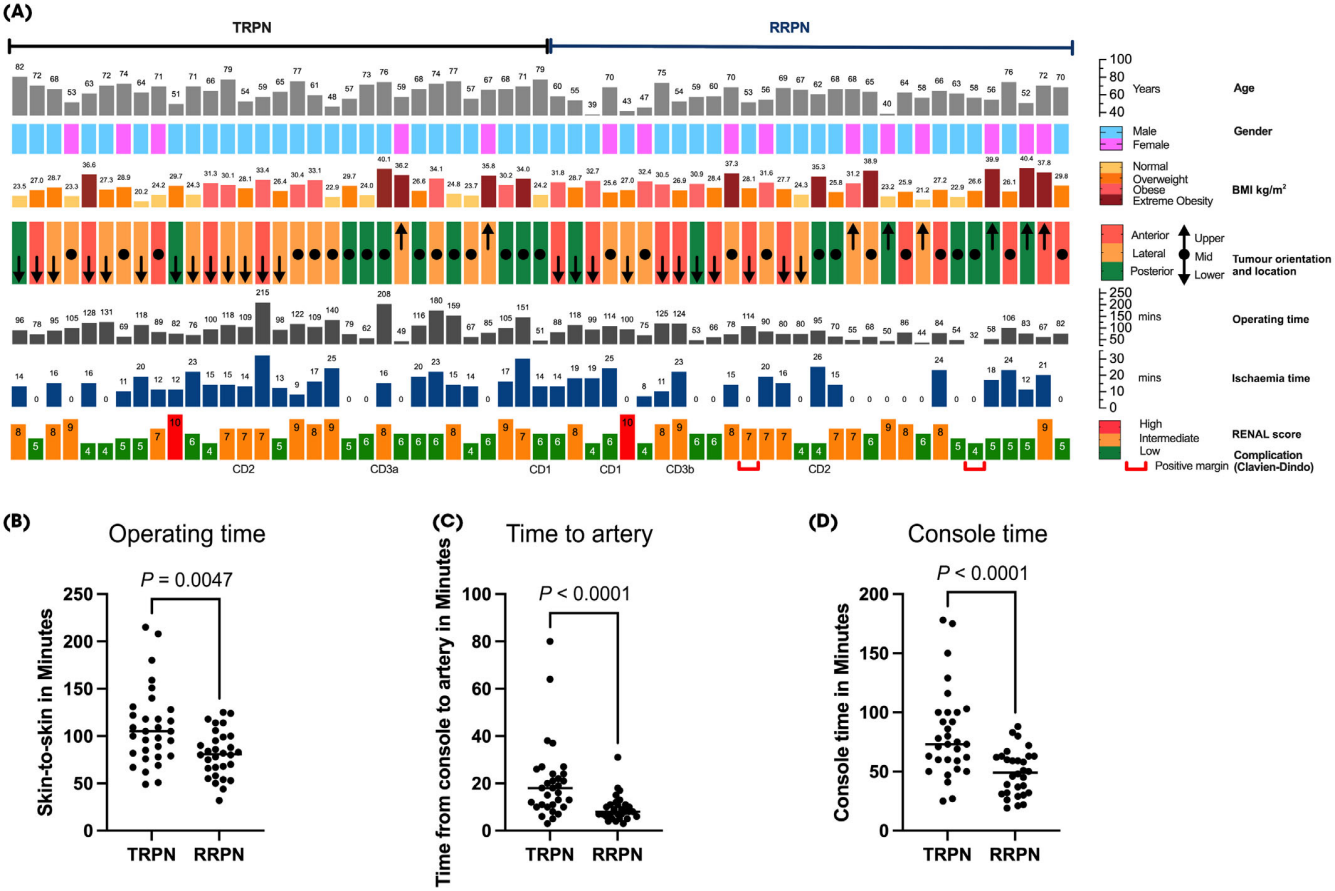
(**A**) Patient and tumour characteristics, operating time and ischaemia time in the transperitoneal robotic partial nephrectomy (TRPN) and retroperitoneal robotic partial nephrectomy (RRPN) groups. (**B**) Scatter plot comparing operating times (min) between the RRPN and TRPN groups. (**C**) Scatter plot comparing time from start on the console to the artery being prepared for clamping (min) between the RRPN and TRPN groups. (**D**) Scatter plot comparing the console times (min) between the RRPN and TRPN groups. BMI, body mass index.

Between September 2022 and November 2023 a total of 98 patients were screened based on the inclusion and exclusion criteria. Of 67 eligible patients, 64 participated in the study and were randomised. This represents an accrual rate of 95.5%. A total of 31 patients were allocated to the transperitoneal surgical approach (TRPN group) and 30 patients to the retroperitoneal approach (RRPN group; Figs 1A and S1). No important changes were made to the methods or outcomes (such as eligibility criteria) after trial commencement. Patient characteristics are shown in detail in Table 1.

**Table 1:** Baseline characteristics.

	Overall (*n* = 61)	RRPN (*n* = 30)	TRPN (*n* = 31)
**Patient demographics**			
Age, years, median (IQR)	65 (57–71)	61 (55–68)	68 (59–74)
Female, *n* (%)	15 (25)	10 (33)	5 (16)
BMI, kg/m^2^, median (IQR)	29 (26–33)	29 (26–33)	29 (24–33)
ASA score, *n* (%)			
1	1 (2)	0 (0)	1 (3)
2	32 (52)	18 (60)	14 (46)
3	25 (41)	11 (37)	14 (46)
4	3 (5)	1 (3)	2 (6)
**Tumour characteristics**			
Left sided tumour, *n* (%)	29 (48)	18 (60)	11 (36)
Orientation, *n* (%)			
Anterior	17 (28)	12 (40)	5 (16)
Lateral	25 (41)	9 (30)	16 (52)
Posterior	19 (31)	9 (30)	10 (32)
Pole, *n* (%)			
Upper	8 (13)	6 (20)	2 (6)
Middle	29 (48)	13 (43)	16 (52)
Lower	24 (39)	11 (37)	13 (42)
Tumour size in mm, median (IQR)	26 (17–37)	25 (17–34)	27 (17–38)
RENAL score, median (IQR)	6 (5–8)	6 (5–8)	6 (5–8)
RENAL complexity, *n* (%)			
Low (4–6)	32 (52)	16 (53)	16 (52)
Moderate (7–9)	28 (46)	13 (43)	13 (42)
High (10–12)	2 (3)	1 (3)	1 (3)
Endophytic, *n* (%)	2 (3)	1 (3)	1 (3)
>50% Exophytic, *n* (%)	37 (61)	18 (60)	19 (61)
<50% Exophytic, *n* (%)	22 (36)	11 (37)	11 (36)
pT stage, *n* (%)			
pT1a	33 (54)	15 (50)	18 (58)
pT1b	10 (16)	6 (20)	4 (13)
pT3a	1 (2)		1 (3)
Benign tumour, *n* (%)	17 (28)	9 (30)	8 (26)

ASA, American Society of Anesthesiologists; BMI, body mass index; IQR, interquartile range; RRPN, retroperitoneal robotic partial nephrectomy; TRPN, transperitoneal robotic partial nephrectomy.

### Peri‐operative Outcomes

In comparison to TRPN, RRPN was associated with significantly shorter operating time (RRPN vs TRPN: median [IQR] 81 [64–99] min vs 105 [79–128] min; *P* = 0.005), console time (RRPN vs TRPN: median [IQR] 49 [32–63] vs 73 [59–100] min; *P* < 0.001) and time from console to artery preparation (RRPN vs TRPN: 7.5 [5–7.5] min vs 18 [10–24] min; *P* < 0.001). The median (IQR) time from skin incision to the start of surgery on the console was significantly shorter in the TRPN group (RRPN vs TRPN: 16 [13–20] min vs 12 [10–15] min; *P* = 0.001 [Fig. 1]). Comparing operating times dependent according to tumour orientation (anterior vs posterior/lateral), RRPN showed a statistically significant time advantage for posterior and lateral tumours (*n* = 44) compared to TRPN (RRPN 73 [54–91] min; TRPN 105 [78–124] min; *P* = 0.003). For anterior tumours, the time advantage of RRPN did not show a statistical significance (RRPN 87 [81–122] min; TRPN 100 [84–172] min; *P* = 0.279).

There was no statistically significant difference in warm ischaemia time (RRPN 11.5 [0–19] min; TRPN 14 [9–17] min; *P* = 0.370), estimated blood loss (RRPN 90 [50–113] mL; TRPN 100 [50–200] mL; *P* = 0.328) or change in haemoglobin level (RRPN −1.7 [−2.4 to −1] g/dL; TRPN −1.8 [−2.4 to −1.2] g/dL; *P* = 0.675).

Histopathological analysis revealed two PSMs in the RRPN group (6.7%; 95% CI 1.9%–21.3%) and none in the TRPN group, with no statistically significant difference (*P* = 0.144). One PSM was identified in a 1.2‐cm well differentiated clear‐cell RCC and the other in a 5.7‐cm chromophobe RCC.

The surgical approach had no significant impact on postoperative acute kidney injury, independent of tumour complexity. The median (IQR) change in estimated GFR was −1 (−10 to +3) mL/min after RRPN and −3 (−11 to +1) mL/min after TRPN (*P* = 0.475; Table 2).

**Table 2:** Peri‐operative outcomes and morbidity.

	Overall (*n* = 61)	RRPN (*n* = 30)	TRPN (*n* = 31)	*P* value
**Peri‐operative outcomes**				
Operating time, min, median (IQR)	89 (70–115)	81 (64–99)	105 (79–128)	0.005* ^,^ ^‡^
Time to console, min, median (IQR)	14 (12–17)	16 (13–20)	12 (10–15)	0.001* ^,^ ^‡^
Time on console, min, median (IQR)	60 (43–80)	49 (32–63)	73 (59–100)	<0.001* ^,^ ^‡^
Time console to artery, min, median (IQR)	11 (7–19)	7.5 (5–7.5)	18 (10–24)	<0.001* ^,^ ^‡^
Ischaemia time, min, median (IQR)	14 (0–19)	11.5 (0–19)	14 (9–17)	0.370^‡^
Complete arterial ischaemia, *n* (%)	36 (59)	13 (43)	23 (74)	0.034* ^,^ ^†^
Incomplete arterial ischaemia, *n* (%)	6 (10)	5 (17)	1 (3)	0.033* ^,^ ^†^
No ischaemia, *n* (%)	19 (31)	12 (40)	7 (23)	0.084^†^
Early unclamping, *n* (%)	11 (18)	4 (13)	7 (23)	0.843^†^
Use of haemostatics, *n* (%)	34 (56)	15 (50)	19 (61)	0.375^†^
PSMs, *n* (%)	2 (3.2)	2 (6.7)	0 (0)	0.144^†^
**Peri‐operative blood loss**				
Preoperative anaemia, *n* (%)	5 (8)	1 (3)	4 (13)	0.173^†^
Preoperative haemoglobin, g/dL (IQR)	15.0 (14–16)	15 (14–16)	15 (13–16)	0.299^‡^
Postoperative Day 1 haemoglobin, g/dL (IQR)	13 (12–14)	13 (12–15)	13 (12–14)	0.330^‡^
ΔHaemoglobin, g/dL (IQR)	−1.7 (−2.4/−1.1)	−1.7 (−2.4/−1)	−1.8 (−2.4/−1.2)	0.675^‡^
Blood loss, mL (IQR)	100 (50–200)	90 (50–113)	100 (50–200)	0.328^‡^
**Peri‐operative kidney function**				
Preoperative CKD stage, *n* (%)	42 (69)	16 (53)	26 (84)	0.010* ^,^ ^†^
I	31 (50.1)	11 (36.7)	20 (64.5)	
II	7 (11.5)	3 (10)	4 (12.9)	
III	3 (4.9)	1 (3.3)	2 (6.5)	
IV	1 (1.6)	1 (3.3)	0 (0)	
Preoperative estimated GFR, mL/min, median (IQR)	85 (74–95)	86 (75.8–98.5)	85 (63–88)	0.123^‡^
Postoperative estimated GFR, mL/min, median (IQR)	78 (62–91)	87 (61.8–99.8)	78 (60–83)	0.053^‡^
ΔEstimated GFR, mL/min (IQR)	−2 (−10.5/+2)	−1 (−10/+3)	−3 (−11/+1)	0.475^‡^
Postoperative AKI, *n* (%)				
Stage I	6 (9.8)	3 (10)	3 (9.7)	0.951^‡^
Stage II	1 (1.6)	0 (0)	1 (3.2)	
Stage III	1 (1.6)	1 (3.3)	0 (0)	
**Peri‐operative morbidity**				
Median (IQR) average NRS pain score				
Postoperatively	1.5 (0–2.8)	1.5 (0–2.6)	1.8 (0.2–3)	0.509^‡^
Postoperative Day 1	1.5 (1–2.5)	1.5 (1–2.4)	1.5 (1–2.5)	0.739^‡^
Postoperative Day 2	1.0 (0.5–2)	1.0 (0.5–2)	1.0 (0.5–2.5)	0.812^‡^
Postoperative Day 3	0.5 (0–1.5)	0.5 (0–1.3)	0.5 (0–1.5)	0.611^‡^
Discharge	0.3 (0.6)	0.4 (0.6)	0.3 (0.6)	0.147^‡^
Pain management				
Metamizole, cumulative g, median (IQR)	4.0 (4.6–5.8)	4.5 (4.4–6.3)	4.0 (4.2–5.7)	0.508^‡^
Additional oxycodone, *n* (%)	5/61 (8)	2/30 (7)	3/31 (10)	0.668^†^
Time to first defaecation, days (IQR)	2 (0–2)	2 (1–2)	2 (0–2)	0.821^‡^
Need for laxatives, *n* (%)	46 (75)	18 (60)	28 (90)	0.006* ^,^ ^‡^
Median (IQR) LOS, days	5 (5–6)	5 (5–5)	5 (5–6)	0.138^‡^
JH‐HLM score 8/24 h (250 + feet)	45 (74)	23 (77)	22 (71)	0.613^†^

Anaemia was defined as haemoglobin <130 g/L for men and <120 g/L for women. AKI and CKD were classified according to the KDIGO (Kidney Disease: Improving Global Outcomes) system.

AKI, acute kidney injury; CKD, chronic kidney disease; IQR, interquartile range; JH‐HLM, John Hopkins Highest Level of Mobility; LOS, length of inpatient stay; NRS, numeric rating scale; PSM, positive surgical margin; RRPN, retroperitoneal robotic partial nephrectomy; TRPN, transperitoneal robotic partial nephrectomy.

* Indicates statistical significance (*P* < 0.05).

^†^ Chi‐squared test.

^‡^ Mann–Whitney *U*‐test.

### Complication Rates at 90‐day Follow‐up

In total, there were no significant differences in terms of complications between the RRPN and TRPN groups. In the entire cohort (*n* = 61), six complications occurred: three (10% [95% CI 3.5%–25.6%]) in the RRPN group and three (9.7% [95% CI 3.6%–24.9%]) in the TRPN group (*P* = 0.955).

One grade I complication occurred in the TRPN group; a patient experienced a transient ischaemic attack and was treated for atrial fibrillation. In both groups, a random finding of minor pulmonary artery embolism was detected on CT and treated with oral anticoagulants for 3 months (grade II). In the RRPN group, a grade II complication was documented due to a re‐admission with fever and small perirenal haematoma; the patient was treated with i.v. antibiotics. In the TRPN group, one grade IIIa complication occurred intra‐operatively due to pleural leakage, requiring a chest drainage. One open revision was needed in the RRPN group (grade IIIb) due to a perirenal haematoma with gross haematuria (Tables 3 and S2).

**Table 3:** Overview of complications within 90 days, classified according to the Clavien–Dindo system.

Complications at 90 days	Patients, *n* (%)		*P* value
	RRPN (*n* = 30)	TRPN (*n* = 31)	
No complications	27 (90)	28 (90)	0.955*
Grade I	0	1	
Grade II	2	1	
Grade IIIa	0	1	
Grade IIIb	1	0	
Complications by severity			0.967*
Minor complications	2 (6.7)	2 (6.5)	
Major complications	1 (3)	1 (3)	

Minor complications were defined as Clavien–Dindo grade ≤ 2 and major complications as Clavien–Dindo grade > 2.

RRPN, retroperitoneal robotic partial nephrectomy; TRPN, transperitoneal robotic partial nephrectomy.

* Mann–Whitney *U*‐test.

### Postoperative Morbidity

**Fig. 2:**
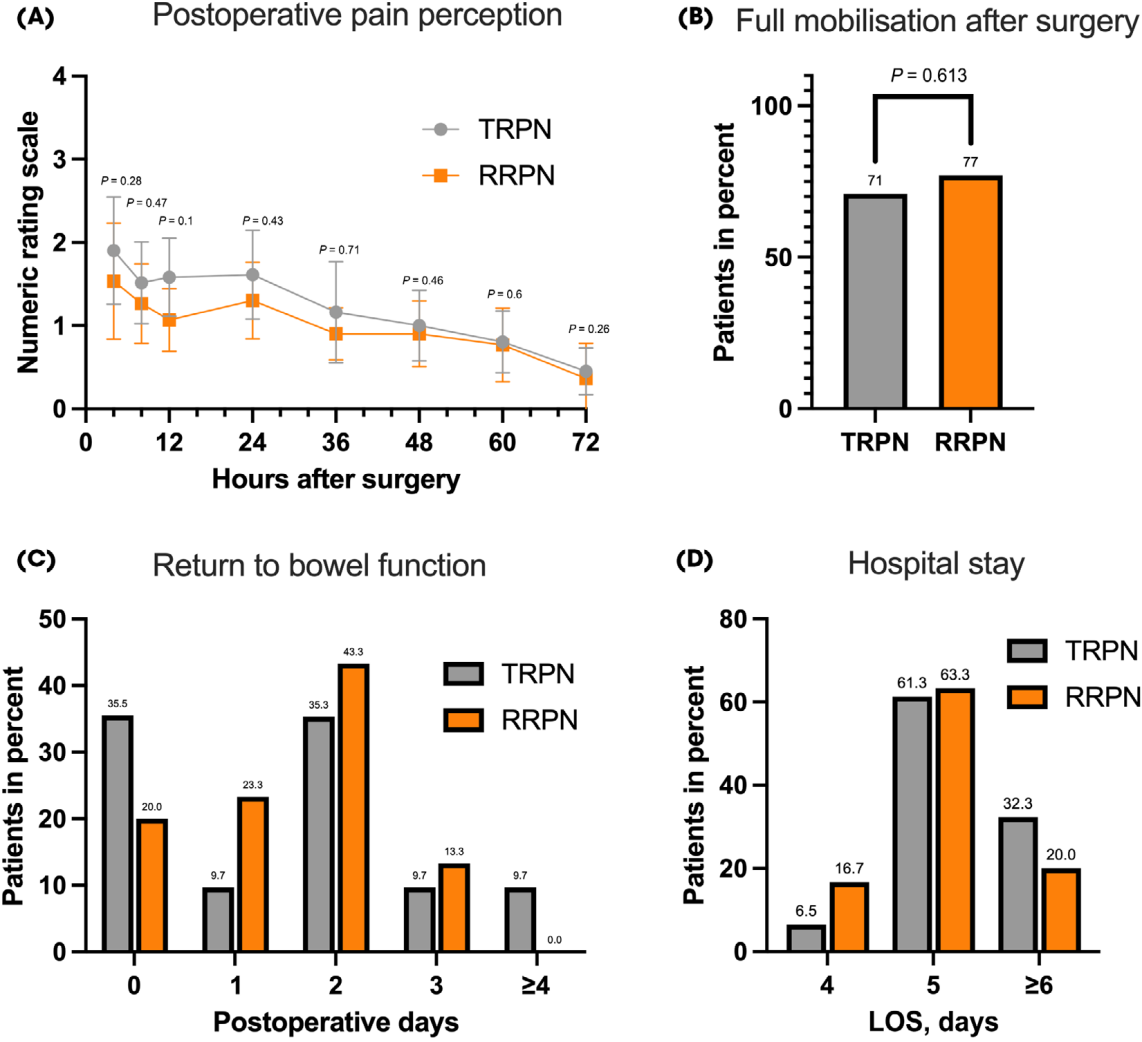
(**A**) Median pain perception within the first 72 h after surgery in the retroperitoneal robotic partial nephrectomy (RRPN) vs the transperitoneal robotic partial nephrectomy (TRPN) group. (**B**) Day on which patients were fully mobilised (defined as John Hopkins Highest Level of Mobility score 8) after surgery in the RRPN vs the TRPN group. (**C**) Day on which patients emptied their bowel for the first time after surgery in the RRPN vs the TRPN group. (**D**) Length of inpatient stay (LOS) after surgery of the patients in the RRPN vs the TRPN group.

Postoperative data are shown in Table 2. Full mobilisation (JH‐HLM score 8) was achieved within the first 24 h after surgery in 28 and 29 patients in the TRPN and RRPN groups, respectively (71% vs 77%; *P* = 0.613 [Fig. 2B]).

Pain was quantified using the NRS. The median NRS score at different timepoints during the postoperative course was lower in the patients who underwent RRPN compared to those who underwent TRPN (Fig. 2A and Table 2). In addition, the median cumulative intake of metamizole did not show a significant difference (*P* = 0.508). Concerning additional opioid analgesics, five patients requested additional oral oxycodone (three vs two patients in the TRPN vs RRPN group, respectively; *P* = 0.668 [Table 2]). Return to bowel function was achieved in all patients in the RRPN group and in 90% of patients in the TRPN group within the first 3 days after surgery (*P* = 0.315; Fig. 2C). In addition, the TRPN group received significantly more laxative medication compared to the RRPN group (90% vs 60%; *P* = 0.006). Most patients received this laxative medication on the second postoperative day after TRPN (68%). In terms of LOS, more patients in the RRPN group were discharged within the first 5 days after surgery compared to the TRPN group (80% vs 67.8%; *P* = 0.138 [Fig. 2D]).

## Discussion

In this single‐centre, open‐label, investigator‐initiated, interventional feasibility RCT (ROPARN study) we compared RRPN with TRPN for localised renal tumours. This study provides the first data from an RCT comparing TRPN with RRPN. From a feasibility perspective, we demonstrated that patients were willing to participate in the study, as verified by an accrual rate of 95.5%. Overall, we showed that RRPN was associated with intra‐operative and postoperative advantages compared to TRPN, with similar PSM and complication rates.

Our first finding, that RRPN was associated with shorter operating times (median time 81 vs 105 min; *P* = 0.005 [Fig. 1B]), is in line with the existing literature. For example, Harke et al. [10] reported median operation durations of 119 min (RRPN) and 139 min (TRPN) in a multicentre matched‐pair analysis of 754 patients. In addition, a systematic meta‐analysis including 21 retrospective studies showed a superiority in mean operating time of 16.7 min in favour of RRPN [21].

Interestingly, this time advantage of RRPN compared to TRPN lost statistical significance for anterior tumours (RRPN vs TRPN: median [IQR] 87 [81–122] min vs 100 [84–172] min; *P* = 0.279) compared to posterior and lateral tumours (RRPN vs TRPN: median [IQR] 73 [54–91] min vs 105 [78–124] min; *P* = 0.003).

The preparation of the renal artery is a fundamental step in nephron‐sparing surgery. The retroperitoneal laparoscopic approach provides quick access to the renal artery because of its dorsal anatomical location. Time from console to artery was significantly shorter in the RRPN compared to the TRPN group (7.5 vs 18 min; *P* < 0.001 [Fig. 1C]). The time between skin incision and artery preparation was shorter in the RRPN compared to the TRPN group, although this was not significantly different (24 vs 30 min; *P* = 0.52). We also found that console time taken to perform RRPN was significantly shorter compared to TRPN (49 vs 73 min; *P* < 0.001 [Fig. 1D]). Similar results were observed in retrospective cohort studies. Mittakanti et al., for example, reported a shorter console time for RRPN (122 vs 141 min; *P* < 0.001) [22].

Although RRPN has some important advantages due to the quick access it provides to the renal surface and hilum, its disadvantages, such as the limited space for port placement and thus greater potential for clashing of robotic arms, should also be noted. Given these disadvantages, it would be reasonable to assume that the PSM and complication rates associated with RRPN would be higher compared to TRPN, however, no significant difference in PSM rate (RRPN 6.7% vs TRPN 0%; *P* = 0.144) was observed in our study. In the previous literature, PSM rates range from 2.8% to 16% for RRPN and from 3% to 12% for TRPN [10, 15, 23].

Before the start of this study, the RRPN surgeon (R.M.) had performed 30 RRPNs. Interestingly, throughout the study we observed an ongoing learning curve for RRPN when comparing the first and second 15 patients. For median operating time (90 vs 68 min; *P* = 0.010), time to reach the artery (33 vs 20 min; *P* < 0.001) and time to reach the console (20 vs 14 min; *P* = 0.009) we observed statistically significant time improvements. This shows the potential of RRPN and implies a possible underestimation of our time benefits.

We did not observe a significant difference in median (IQR) blood loss between the two surgical approaches (RRPN 90 [50–113] mL vs TRPN 100 [50–200] mL; *P* = 0.328). In contrast to our findings, Mjaess et al. (161 vs 293 mL; *P* < 0.001) and Mittakanti et al. (*P* < 0.001) reported significantly lower estimated blood loss in the RPPN compared to the TRPN group [15, 22].

Regarding complication rates (RRPN 10% vs TRPN 10%; *P* = 0.966), our series is comparable to other studies, such as the study by Mittakanti et al., in which complication rates were also similar in the two groups (RRPN 9.3% vs TRPN 8.7%; *P* = 0.88) [22].

In our study, one grade IIIa complication occurred intra‐operatively while performing TRPN on a tumour with a posterior location. After pleural leakage, a chest drain had to be inserted for several days. On the other hand, a grade IIIb complication occurred after RRPN for an anterior tumour, with the need for open revision for postoperative bleeding (Tables 3 and S2). Harke et al. [10] reported more complications in their TRPN group (RRPN 14% vs TRPN 22%; *P* = 0.007), including major complications in 3.4% vs 8.5% of patients (*P* = 0.06). By contrast, Choo et al. [24] observed more complications in their RRPN group (RRPN 14% vs TRPN 9.1%; *P* = 0.430).

In this RCT we showed that the RRPN group had less postoperative pain on average, as well as a higher percentage of patients with full mobilisation (77% vs 71%; *P* = 0.613) within the first 24 h, and faster bowel canalisation after surgery. In their recent study, Bertolo et al. [14] investigated these issues using a retrospective propensity‐score‐matched analysis of RRPN and TRPN. They reported that patients who had undergone TRPN requested significantly more additional NSAIDs and opioids compared to patients who had undergone RRPN (NSAIDs 26% vs 9% [*P* = 0.004]; opioids 11% vs 0% [*P* = 0.002]). The median proportions of patients given paracetamol was similar in the two groups (RRPN 5.5% vs TRPN 5%; *P* = 0.7). Pain quantified based on median visual analogue scale score at discharge was not significantly different between the groups (median [IQR] score: 1 [0–3] vs 2 [1–3]; *P* = 0.07) [14]. In our study, we observed a lower median pain perception score in the postoperative course in the RRPN compared to the TRPN group (Fig. 2A). Administration of metamizole was not significantly different between the two groups, while opioids were administered less frequently in the RRPN compared to the TRPN group (Table 2).

In all patients in the RRPN group return of bowel function occurred within the first 3 days, compared to 90% of patients in the TRPN group (Fig. 2C), and the RRPN group received significantly less laxative medication than the TRPN group (90% vs 60%; *P* = 0.006). Bertolo et al. [14] reported a median time to bowel canalisation of 3 days in both groups. In comparison, Porpiglia et al. reported a longer median (IQR) time to canalisation (3 [2–5] vs 2 [1–3] days; *P* < 0.0001) after TRPN compared to RRPN [25].

Regarding the LOS, we showed that more patients in the RRPN group were discharged within the first 5 days after surgery compared to the TRPN group (80% vs 67.8%; *P* = 0.277 [Fig. 2D]).

While no significant difference was observed in LOS, this may be attributable to our standard practice, which is to discharge patients 4 days postoperatively. This is longer than the median stay described in other studies, which varies between 1 and 3 days. Hughes Hallet et al. (2.5 vs 4.6 days; *P* < 0.001), Porpiglia et al. (2 vs 3 days; *P* < 0.0001) and Mittakanti et al. (1.7 vs 1.9 days; *P* = 0.006) showed significantly shorter LOS after RRPN [22, 25, 26].

Reduced operating time and shorter LOS after RRPN could explain the cost savings associated with RRPN compared to TRPN that were reported in a bicentre study by Laviana et al. [27], emphasising the importance of reduced peri‐operative morbidity.

As the superior morbidity results in the RRPN group could be attributable to an imbalance between the groups in patient characteristics or tumour characteristics, we compared the two groups statistically. No significant differences were found in gender distribution (*P* = 0.119), American Society of Anesthesiologists score (*P* = 0.363), body mass index (*P* = 0.516), tumour size (*P* = 0.341), tumour orientation (*P* = 0.2) or RENAL score (*P* = 0.737). The only significant difference observed was in mean age, which was higher in the TRPN group (mean age 67 vs 61 years; *P* = 0.009). Superior morbidity results after RRPN have already been described in several retrospective studies. Porpiglia et al. [25] reported a slightly lower intra‐operative complication rate, with earlier postoperative recovery for retroperitoneal minimally invasive surgery (shorter drainage duration and LOS: 3 vs 2 days for both variables; *P* < 0.0001). In addition, Sharma et al. [28] reported significantly higher trifecta outcomes after RRPN (70.2% vs 53%; *P* < 0.001).

These findings of less morbidity could be attributable to the shorter operating time, absence of need for bowel mobilisation, and less kidney rotation depending on tumour location. Carbonara et al. [16], however, reported similar postoperative and functional outcomes for patients with posterolateral renal tumours for RRPN and TRPN.

As evidence of the superiority of RRPN in selected cases grows, greater dissemination of the retroperitoneal approach should be encouraged to exploit its benefits. Ideally, most surgeons should be able to perform partial nephrectomy using both approaches. As RRPN entails a learning curve, surgeons experienced in TRPN should be encouraged to implement the retroperitoneal approach. For a skilled TRPN surgeon, successful implementation of RRPN is possible after 30 cases [29]. This will facilitate more and larger multicentre RCTs.

This study has some limitations. First, owing to the relatively small sample size and single‐centre setting, no conclusive confirmatory testing could be performed. In addition, for rarely occuring adverse outcomes, such as PSM rate and complications, the broad range of the 95% CIs demonstrates the uncertainty of the estimator, and these results must be interpreted with caution. Some endpoints, such as LOS, depend on local workflows and should also be interpreted with caution. In our hospital, the standard for patient discharge after partial nephrectomy is Day 4. In our study, all RRPNs were performed by one surgeon (R.M.), while the TRPNs were performed by one of three surgeons (S.D., J.B., R.M.), which confers a potential bias. As RRPN was associated with a significantly shorter operating time and was performed by only one surgeon (R.M.), it could be assumed that the RRPN surgeon was *per se* faster than the TRPN surgeons. To address this issue, we compared the median TRPN operating times between the RRPN surgeon (R.M.) and the other surgeons (S.D., J.B.) and found no significant difference (96 min vs 109 min; *P* = 0.142). It should be noted that this study included not only tumours that were conveniently located for RRPN, but also tumours with an unfavourable anterior location. However, we acknowledge that highly complex or extremely anterior tumours were not included, which could have been a confounding factor.

In conclusion, our study demonstrates the feasibility of trial recruitment in an RCT setting for TRPN and RRPN. We showed that RRPN was significantly faster and the time taken for artery identification was significantly shorter compared to TRPN, without significant increases in complication and PSM rates. Time benefits were further increased for posterior tumours accessed via a retroperitoneal approach. In the postoperative setting, patients undergoing RRPN had less pain, mobilised quicker and had fewer problems with bowel canalisation. RRPN is a safe alternative to TRPN and both approaches should be mastered and offered by experienced RPN surgeons.

We conclude that RRPN should be the first choice for posterior and lateral renal tumours. In patients with anterior tumours TRPN is more suitable than RRPN, except in patients with extensive prior abdominal surgery. Further RCTs in a multicentre setting are necessary to strengthen the evidence of this study.

## Disclosure of Interests

The authors acknowledge they have no conflict of interest.

## Funding

No specific funding was obtained for this study.

## Supporting information


**Fig. S1.** Consolidated Standards of Reporting Trials (CONSORT) flow diagram.
**Table S1.** Detailed overview of excluded patients.
**Table S2.** Detailed overview of complications within hospital and after discharge within 90 days, classified by Clavien Dindo.
