# Supplementary material for: Randomised controlled feasibility trial of retroperitoneal vs transperitoneal robot‐assisted partial nephrectomy: the ROPARN study
Source: BJU Int. 2025 Jan 22;135(6):977–86. doi: 10.1111/bju.16653 (PMC12053051; doi:10.1111/bju.16653)
Supplement: Supplementary file 1 — Fig. S1. Consolidated Standards of Reporting Trials (CONSORT) flow diagram. Table S1. Detailed overview of excluded patients. Table S2. Detailed overview of complications within hospital and after discharge within 90 days, classified by Clavien Dindo. [file BJU-135-977-s001.doc]

**Figure S1: Consolidated Standards of Reporting Trials (CONSORT) flow diagram.**

Assessed for eligibility (n = 98)

Excluded (n = 34)

Not meeting inclusion criteria

(n = 28)

Refused to participate

(n = 3)

Other reasons (n = 3)

**Enrollment**

Randomized (n = 64)

#

**Allocation**

**Follow up**

**Analysis**

Randomized to TRPN

(n = 32)

Received allocated intervention (n = 31)

Did not receive allocated intervention (n =1)

(refused surgery)

Randomized to RRPN

(n = 32)

Received allocated intervention (n =30)

Did not receive allocated intervention (n = 2)

(1 refused surgery, 1 underwent open PN)

Lost to 90d-follow up (n = 0)

Lost to 90d-follow up (n = 0)

Analyzed (n = 31)

Analyzed (n = 30)

**Table S1: Detailed overview of excluded patients.**

| **Number** | **Cause of exclusion** |
| --- | --- |
| 9 | Combination of tumor characteristics complicating surgery (e.g. completely endophytic, renal pelvis infiltration, contact to renal artery/vein, size) with possible disadvantage for patient being randomized to RRPN. |
| 6 | Combination of tumor orientation (e.g. complex anterior, anterior upper pole) and characteristics (e.g. size/endophytic) with possible disadvantage for patient being randomized to RRPN. |
| 5 | Patients with a history of extensive transabdominal or retroperitoneal surgery |
| 1 | Double kidney with ureter fissus and tumor contact to renal hilum |
| 2 | vulnerable patients (with cognitive impairments (e.g. dementia)) |
| 1 | Impaired mobilization before surgery |
| 3 | Multiple tumors |
| 1 | Recurrent tumors |
| 3 | Refused to participate |
| 3 | RRPN surgeon not present |
| **Σ28** | **Not meeting inclusion criteria** |
| **Σ3** | **Refused to participate** |
| **Σ3** | **Other reasons** |

**Table S2: Detailed overview of complications within hospital stay and after discharge**

**within 90 days, classified by Clavien Dindo.**

| **Complications at 90 d** | **Patients, n (%)** | | **p-value** |
| --- | --- | --- | --- |
|  | RRPN (n=30) | TRPN (n=31) |  |
| **No complications** | 27 (90) | 28 (90) | 0.966 ^Chi^ |
| **Within hospital stay** | 1 (3.3%) | 2 (6.5%) | 0.573^Chi^ |
| Grade I |  | 1 (1.6%)  transient ischaemic attack with dysarthria on the first day postop. gone after 24 hours without intervention. |  |
| Grade II | 1 (1.6%)  minor periphere pulmonary artery on the first day postop. NOAK for 3 months. |  |  |
| Grade IIIa |  | 1 (1.6%)  Thorax drainage needed after pleural leakage. |  |
| Complications by severity |  |  |  |
| Minor complications | 1 (1.6%) | 1 (1.6%) | 0.981^Chi^ |
| Major complications | 0 | 1 (1.6%) | 0.321^Chi^ |
| **After discharge** | 2 (7%) | 1 (3%) | 0.574^Chi^ |
| Grade II | 1 (1.6%)  admission with fever for intravenous antibiotics. CT graphic small perirenal hematoma. No further intervention needed. | 1 (1.6%)  random finding of pulmonary artery embolism. 30 days after surgery. NOAK for three months. |  |
| Grade IIIb | 1 (1.6%)  perirenal hematoma and macrohematuria. Need of transfusion and open revision with urethral stenting for 6 weeks. |  |  |
| Complications by severity |  |  |  |
| Minor complications | 1 (1.6%) | 1 (1.6%) | 0.981^Chi^ |
| Major complications | 1 (1.6%) | 0 | 0.315^Chi^ |
| NOAK = new oral anticoagulation; RRPN = retroperitoneal robotic partial nephrectomy; TRPN = transperitoneal robotic partial nephrectomy.  ^MW^ Mann-Whitney U test  ^Chi^ χ^2^ test.  Minor complications = Clavien Dindo≤ 2  Major complications = Clavien Dindo>2 | | | |
